# Small punch tensile/fracture test data and 3D specimen surface data on Grade 91 ferritic/martensitic steel from cryogenic to room temperature

**DOI:** 10.1016/j.dib.2016.08.061

**Published:** 2016-09-03

**Authors:** Matthias Bruchhausen, Jean-Marc Lapetite, Stefan Ripplinger, Tim Austin

**Affiliations:** European Commission, Joint Research Centre, Institute for Energy and Transport, 1755 LE Petten, The Netherlands

**Keywords:** Small punch tensile test, Curve data, X-ray computed tomography, Ferritic/martensitic steel

## Abstract

*Raw data from small punch tensile/fracture tests at two displacement rates in the temperature range from* −*196* *°C to room temperature on Grade 91 ferritic/martensitic steel are presented. A number of specimens were analyzed after testing by means of X-ray computed tomography (CT). Based on the CT volume data detailed 3D surface maps of the specimens were established. All data are open access and available from Online Data Information Network (ODIN)*https://odin.jrc.ec.europa.eu*. The data presented in the current work has been analyzed in the research article “*On the determination of the ductile to brittle transition temperature from small punch tests on Grade 91 ferritic-martensitic steel*”* (M. Bruchhausen, S. Holmström, J.-M. Lapetite, S. Ripplinger, 2015) [1].

**Specifications Table**

**Table:** 

Subject area	*Physics*
More specific subject area	*Materials Science*
Type of data	Materials Pedigree Data, Data tables (curve data), 3D surface data
How data was acquired	*The raw data result from small punch tensile/fracture testing according to the current European Code of Practice*[5]*by means of an Instron type 5586 universal testing machine. The CT data were acquired using a Phoenix Nanotom S research edition tomograph. The surfaces determination was carried out by means of the VGStudio Max 2.2 software package from Volume Graphics.*
Data format	*Raw data for curve data (background subtracted), 3D surface data calculated from 3D volume data*
Experimental factors	*The specimens were polished to a surface roughness R*_*a*_*of <0.15* μ*m.*
Experimental features	*Two series of small punch tensile tests were carried out at displacement rates of 0.5* *mm/s, 0.05* *mm/s and 0.005* *mm/s with a punch of 2* *mm diameter. The test temperatures ranged from −196* *°C to room temperature.*
Data source location	*1755 Petten, The Netherlands*
Data accessibility	*The data are available in this article and are hosted at the European Commission materials database at*https://odin.jrc.ec.europa.eu

**Value of the data**

• The small punch testing technique is widely used, but many aspects of the testing procedures and the data evaluation are still under discussion [2], [3], [4]. Openly available data sets allow for different evaluation procedures to be compared on the same raw data.
• The data sets directly allow studying the influence of the punch displacement rate and the temperature on the force-deflection curves from small punch testing. In this study an unusually small punch diameter has been used which allows - through comparison with data from other sources - studying also the impact of the punch geometry.
• The use of X-ray computed tomography for reconstructing the surface of some of the deformed specimens after testing allows a very detailed post-test analysis.

## 1 Data

The data are force-displacement curves of small punch tensile tests at different punch displacement rates and temperatures.

X-ray computed tomography imaging has been used to reconstruct full 3D images of some of the tested specimens. The volume data from the CT analysis have been used to determine 3D surface models which are included in the data sets.

## 2 Experimental design, materials and methods

### 2.1 Material

The particular batch of Grade 91 steel used in the present work as part of the FP7 project MATTER [6] was originally produced for the FP6 EUROTRANS project (domain 4: DEMETRA). Its chemical composition is listed in Table 1. The material was delivered by Industeel, Arcelor group as hot rolled and heat treated plate with a thickness of 15 mm.

The material received a normalization heat treatment of 15 min (1 min for 1 mm thickness) at 1050 °C followed by water cooling to room temperature, which resulted in a fully martensitic structure. During the subsequent tempering treatment the alloy was held for 45 min (3 min for 1 mm thickness) at 770 °C followed by cooling to room temperature in still air.

### 2.2 Test rig and procedure

According to the current Code of Practice [5], during a small punch tensile/fracture test a punch with a hemispherical tip is pushed along its axis of symmetry through the center of a disc shape specimen (of thickness 0.5 mm and diameter 8 mm, surface roughness less than *R_a_*=0.15e−6 m on both sides). All specimens were cut from the same block of material but with two different orientations: TS and TL. The letters L, T and S respectively refer to the longitudinal (rolling), transverse and short transverse directions. The designation of the groups follows the principle that the letter missing in the designations indicates the axis perpendicular to the specimen plane.

During the test the punch is moved at a fixed displacement rate and the force needed to punch through the specimen is measured. The relation between the force and either the displacement of the punch tip or the deflection of the specimen is used to derive basic mechanical material characteristics, such as the ultimate tensile strength and the yield stress [2], [4].

**Fig. 1:**
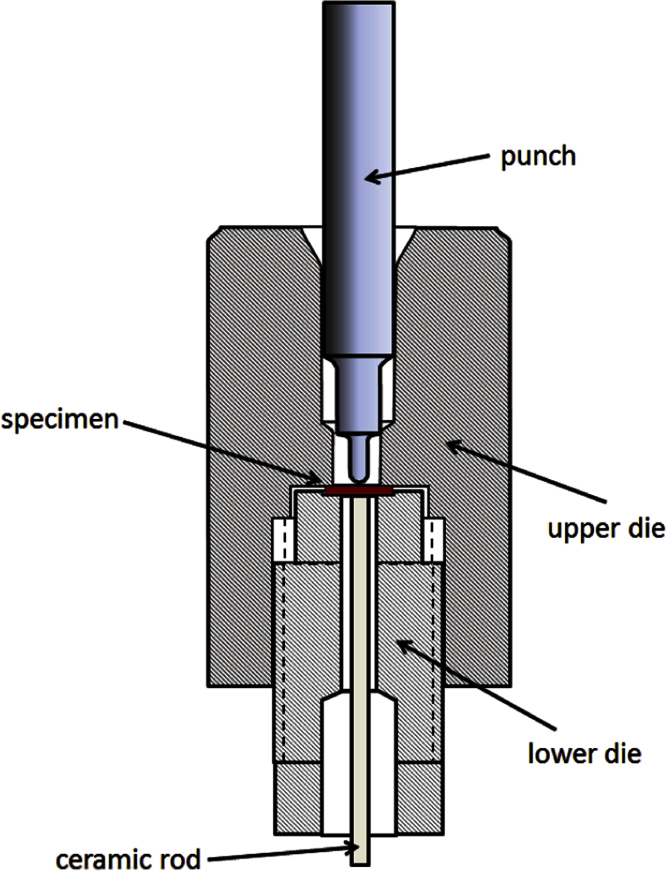
Scheme of the test setup.

The test rig used in the present study is depicted in Fig. 1; the main geometrical dimensions are listed in Table 2. The specimen in the center of the test rig is solidly clamped between two dies which are tightened together by the threaded fitting of the upper and lower die. A ceramic rod touching the specimen from below transfers the deflection to a Linear Variable Displacement Transducer (LVDT) as in the Code of Practice [5]. Measuring the specimen deflection (from below the specimen) avoids errors resulting from the compliance. Because of specimen thinning during the test using the deflection leads to a slight underestimation of the fracture energy.

The entire assembly of dies, specimen and punch (Fig. 1) was positioned in an environmental chamber (Instron 3119-407 series) that can be cooled down to −196 °C by liquid nitrogen and heated up to 300 °C. The displacement of the punch was controlled by an Instron type 5586 universal testing machine. To avoid problems related to misalignment of the push rod and the specimen, the punch was carefully positioned on the specimen before locating the assembly in the environmental chamber. The push rod from the universal testing machine was not rigidly connected to the punch.

Because of the spatial limitations, the LVDT measuring the deflection was mounted inside the environmental chamber. The movement of the ceramic rod touching the specimen from below (Fig. 1) was transferred to the LVDT by means of a cantilever. Verification tests in the absence of the specimen showed agreement between the displacement of the cross head of the testing machine (which was itself confirmed with a laser hologage (Mitutoyo EF 11PRH) and the LVDT signal. Carrying out a series of such verification tests at different temperatures allowed correction factors to be determined, which were then later used to compensate for the temperature influence on the LVDT signal.

The test temperature was controlled by means of a T-type thermocouple (0.5 mm diameter) inserted in the ceramic rod and touching the specimen from below. Additional thermocouples at different locations in the environmental chamber allowed the correct functioning of the equipment to be monitored.

The tests were performed at two cross head displacement rates (0.5 mm/s and 0.005 mm/s). Test temperatures ranged between −196 °C and 23 °C.

A summary of the available data sets is given in Table 3 and references [7], [8], [9], [10], [11], [12], [13], [14], [15], [16], [17], [18], [19], [20], [21], [22], [23], [24], [25], [26], [27], [28].

### 2.3 Post-test specimen data

A nano-CT system (Phoenix Nanotom S 180 from General Electric) was used to examine the SP samples tested with a displacement rate of 0.5 mm/s. The maximum tube voltage is 180 kV and the maximum power is 15 W. It is equipped with an ultra-high performance nanofocus X-ray tube, a Hamamatsu flat panel sensor C7942SK-25, (2304×2304 Pixel, pixel pitch 50 µm). The precision mechanics and the advanced software modules for data analysis offer detail detectability up to 200 nm and a minimum voxel ("volume pixel") size of (500 nm)^3^. Four different operation modes are offered to examine sub-micron applications to applications of high intensity.

To avoid Feldkamp artifacts [29] the samples were placed on a holder with a tilt angle of approximately 45°. Geometrical magnification based on a focus-object distance (FOD) of 17 mm and a focus-detector distance of 200 mm leads to a voxel size of 4.25 µm.

The scans in the present study were carried out with a voltage of 130 kV and a current of 120 mA. A 0.5 mm Sn filter was used to cut off the low-energetic radiation. The modus for high intensity application with an X-ray dependent focal spot size was used. Detector shift function to avoid residual ring artifacts and a pre-scan function for later drift analysis and correction were enabled.

Phoenix datos/x software from GE was used to re-construct the voxel data. The software modules for beam hardening correction, ring artifact reduction and geometrical calibration were applied during the re-construction process.

Metrology software VGStudio Max 2.2 from Volume Graphics was used for the visualization of the volume data file. To enhance the contrast a reduced grey value range was used for the re-construction. Data sets were filtered using median algorithm with a window size of three voxels. First a standard surface determination was carried out during which the specimen surface was reconstructed on the level of voxels. A subsequent advanced mode surface determination step with a search distance of 4 voxels allowed interpolating the surface to a sub-voxel level.

To obtain a spatial calibration, first the diameter of the SP sample was determined by means of a calibrated micrometer gauge. This value was compared to the diameter of a cylinder fitted by the metrology software (1000 fit points) to the specimen circumference. By modifying the voxel size, the diameter determined by the metrology software was then brought to agreement with the micrometer value.

**Fig. 3:**
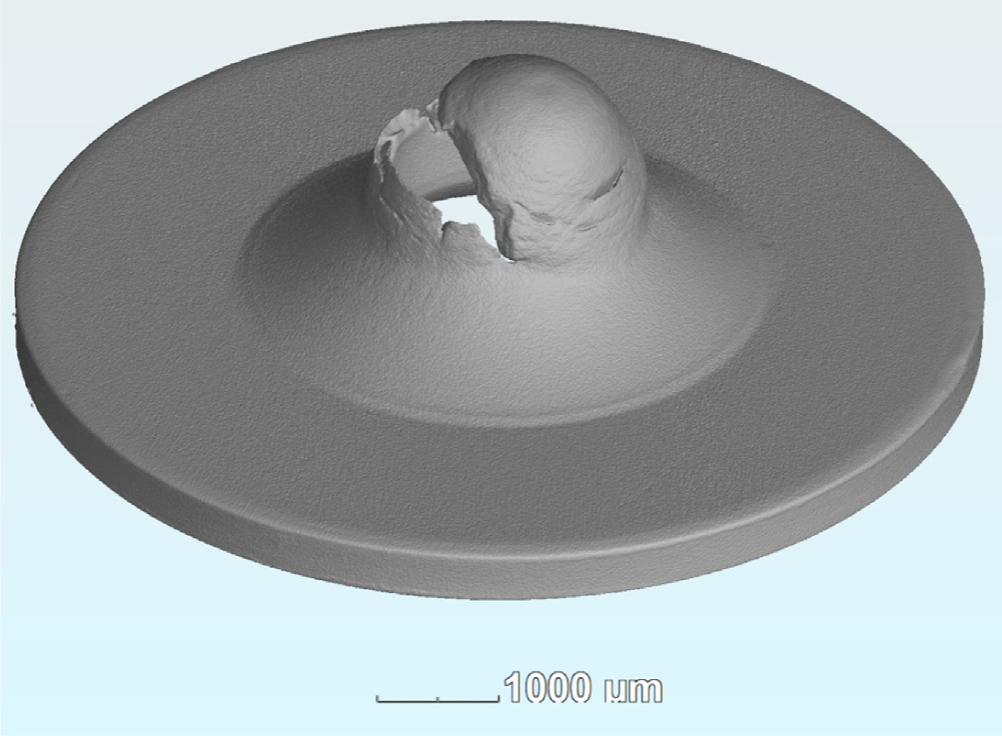
Image of the 3D volume data of specimen TL25 after testing.

The surface data were exported in STereoLithography (.stl) format using the micrometer scale and are available for download. An example reconstructed surface image is shown in Fig. 3.

## Figures and Tables

**Fig. 2:**
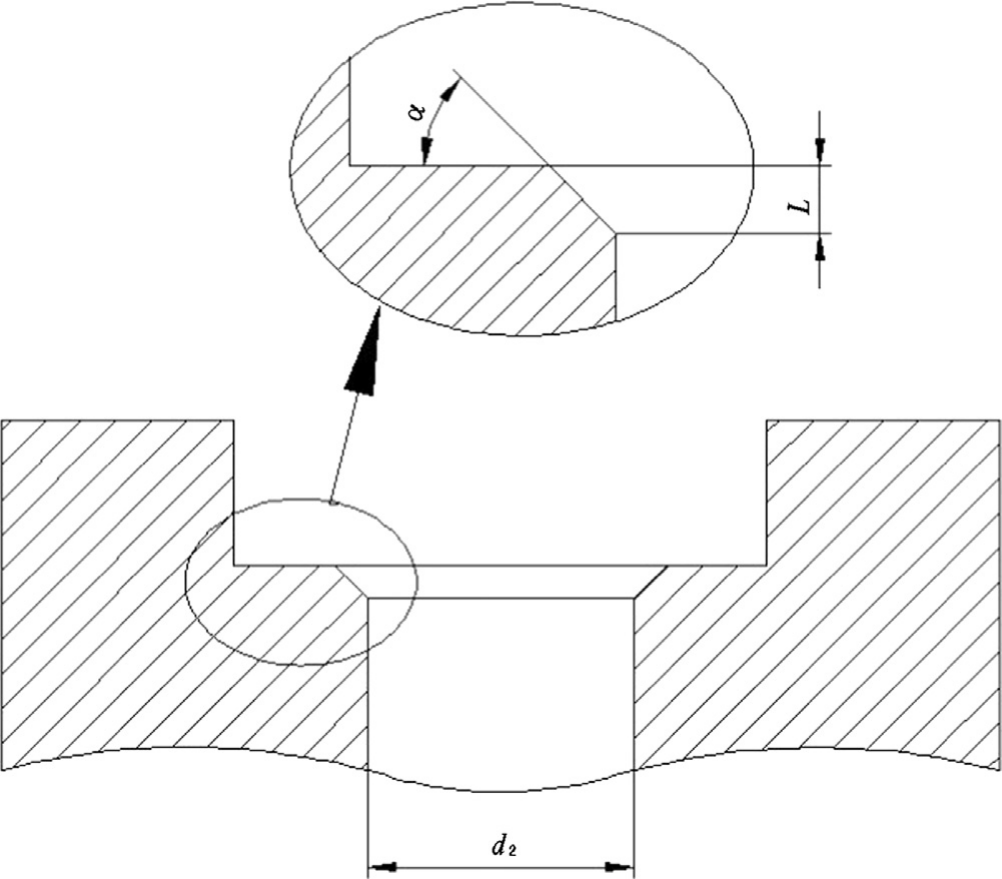
Details of the chamfer.

**Table 1:** Chemical composition of the tested heat in weight percent according to the supplier.

**C**	**Mn**	**P**	**S**	**Si**	**Cu**	**Ni**	**Cr**
0.097	0.386	0.020	0.0005	0.218	0.080	0.115	8.873

**Mo**	**Al**	**Nb**	**V**	**Ti**	**N**	**Fe**	

0.871	0.009	0.077	0.195	0.003	0.0440	bal.	

**Table 2:** Relevant geometric characteristics of the test rig (or parameters relating to the chamfer refer to Fig. 2).

**Feature**	**Symbol**	**Dimension**
Punch tip radius	*r*	1 mm
Diameter of receiving hole (lower die)	*d*_2_	4 mm
Chamfer (lower die)	*l*	0.2 mm
Chamfer angle	*α*	45°
Specimen diameter	*d*_1_	8 mm
Specimen thickness	*h*	0.5 mm

**Table 3:** List of tests (*for test TL10 no curve data is available because of a problem with the data acquisition).

**Test/specimen identifier**	**Displacement rate [mm/s]**	**Temperature [°C]**	**3D data available (Y/N)**
TS08	0.005	−196	N
TS15	0.005	−196	N
TS11	0.005	−170	N
TL08	0.005	−150	N
TL23	0.005	−132	N
TS09	0.005	−120	N
TL17	0.005	−100	N
TL22	0.005	−60	N
TS05	0.005	−20	N
TL24	0.005	23	N
TL11	0.05	−150	N
TL14	0.5	−196	Y
TL10*	0.5	−196	Y
TL16	0.5	−170	Y
TS13	0.5	−170	Y
TL12	0.5	−150	Y
TL20	0.5	−130	Y
TS07	0.5	−120	Y
TL26	0.5	−100	Y
TL18	0.5	−60	Y
TS10	0.5	−20	Y
TL25	0.5	23	Y
