# Supplementary material for: Small punch tensile/fracture test data and 3D specimen surface data on Grade 91 ferritic/martensitic steel from cryogenic to room temperature
Source: Data Brief. 2016 Sep 3;9:245–51. doi: 10.1016/j.dib.2016.08.061 (PMC5021917; doi:10.1016/j.dib.2016.08.061)
Supplement: Supplementary file 1 — Supplementary material [file mmc1.doc]

Petten, 31 May 2016

**DIB-D-16-00352R1**

Dear Editor,

The authors don't have any commercial interest in the data, testing method or other aspects of the submission.

There is no conflict of interest.

M. Bruchhausen
